# Treatment of Skull Base Diseases: A Multidisciplinary Challenge

**DOI:** 10.3390/jcm12041492

**Published:** 2023-02-13

**Authors:** Leonardo Franz, Elisabetta Zanoletti, Piero Nicolai, Marco Ferrari

**Affiliations:** 1 Section of Otorhinolaryngology—Head and Neck Surgery, Department of Neuroscience, “Azienda Ospedale Università di Padova”—University of Padua, 35122 Padua, Italy; 2 Guided Therapeutics (GTx) Program International Scholarship, University Health Network (UHN), Toronto, ON M5G 2C4, Canada; 3 Artificial Intelligence in Medicine and Innovation in Clinical Research and Methodology (PhD Program), Department of Clinical and Experimental Sciences, University of Brescia, 25121 Brescia, Italy; 4 Audiology Unit, Department of Neuroscience, University of Padova, 35122 Treviso, Italy

The skull base has always been regarded as a frontier by surgeons and radiation oncologists since it represents the interface between the intracranial and the extracranial compartment and hosts several critical anatomical structures with an extremely complex and close relationship.

Anatomically, the skull base is not a homogeneous region, since it adjoins different regions (the sinonasal compartment, the middle and inner ear, the upper parapharyngeal space, and the posterior neck and spine); simultaneously, it is crossed by delicate neurovascular structures from the intracranial to the extracranial region.

Historically, the sub-specialties of skull base surgery developed from the disciplines involved in the management of the neighboring structures; contemporary anterior skull base surgery mainly derived from rhinology and lateral skull base surgery as an extension of otology, while posterior skull base surgery and the classical open trans-cranial approaches to the anterior and middle fossae were part of the traditional neurosurgical repertoire. As a result, skull base surgery is currently a multidisciplinary field in which neurosurgeons, otorhinolaryngologists, and maxillofacial and plastic-reconstructive surgeons often cooperate; thus, the skull base is no longer considered a barrier for each of these surgical disciplines but a common field of interest [1].

From a pathological perspective, the skull base may be involved by a spectrum of biologically heterogenous neoplasms. In the anterior skull compartment, tumors may arise from the sinonasal epithelium (both mucosa and glands), as well as soft tissues, bone, and cartilage [2]. Among these histological varieties, there are entities that are characteristic of this site, such as olfactory neuroblastoma and juvenile angiofibroma, which require specific treatment approaches. Tumors of the lateral skull base can either arise primarily from the temporal structures (i.e., the external auditory canal, middle ear, or mastoid) or invade them secondarily [3,4]. Common extratemporal origins include the parotid gland, auricle, and infratemporal fossa (comprising cutaneous, salivary, or mesenchymal histologies), whereas most primary lateral skull base tumors are cutaneous, originating from the skin of the external acoustic canal [5]. This wide heterogeneity complexifies the management of skull base tumors and hinders the development of a single, standardized approach that can be used for all tumors.

Over the past decades, skull base surgery has undergone dramatic development towards reduced invasiveness and enhanced surgical precision, which has occurred alongside the evolution of supporting technology. The advent of microsurgery, along with the endoscopic revolution at the beginning of the 21st century, allowed surgeons to gain access to extremely critical anatomical areas and structures, including the clivus, sella and para-sellar regions, cavernous sinus, petrosal temporal bone up to the petroclival junction, cerebellopontine angle, foramen magnum, cranial nerves, and internal carotid artery [6,7,8,9].

From a surgical perspective, the bony frame of the skull base is neither a barrier nor a constraint for the surgical approach. Rather, it represents a gateway to the critical structures located within the bone of the skull base, since most of the surgical approaches consist in drilling out the bone of the skull base until the target areas are reached. The recent development of virtual surgical planning protocols and image-guided surgery systems have rendered the “surgical corridor” paradigm even more crucial, thus supporting the accurate planning of the surgical trajectory towards the target area and facilitating the anatomical orientation [10,11,12,13,14]. In this setting, augmented reality visualization modalities may represent a new frontier in which to further improve the benefits provided by the image-guided surgery paradigm [12,13,15].

Along with the current endoscopic techniques and novel extended approaches (Figure 1), which continue to facilitate the progression of endoscopy ever onward, a contemporary skull base surgeon should also be aware of the historical development of this surgical field and the basic microsurgical techniques and traditional open approaches with which to fully master the surgery of difficult cranial base lesions and achieve safe and effective treatment (Figure 2 and Figure 3). Simultaneously, it is crucial that a skull base surgeon be predisposed to collaborate with colleagues from other fields, including non-surgical disciplines. This multidisciplinary perspective must be considered a mainstay when approaching diseases of the skull base.

The same anatomical and functional principles guiding surgery should also be applied to radiation oncology. In fact, targeting treatment to the involved areas (while sparing critical structures) represents a major challenge for contemporary skull base radiation oncologists. For most resectable craniofacial malignancies, postoperative radiation has a well-established role. Intensity-modulated radiation therapy still represents the most frequently used radiation technique, although the evidence for innovative and potentially less invasive and/or more effective approaches, such as proton therapy and carbon ion irradiation, is promising. In particular, for radioresistant tumors, or in the case of gross residual disease after surgery, the use of proton and carbon ion therapy is now strongly recommended in view of the possibility of escalating the dose and biological effectiveness towards the target while minimizing the irradiation of neurological structures [17,18] (Figure 4). This concept frequently applies to chordoma, chondrosarcoma, mucosal melanoma, and adenoid cystic carcinoma. All these considerations with respect to postoperative radiation therapy can be further translated to the definitive setting when surgery is contraindicated.

Systemic therapy represents another promising field of multidisciplinary treatment for skull base tumors, especially in an induction/neoadjuvant setting (Figure 5), with the aim of increasing locoregional control, decreasing morbidity inherent to local therapy, and reducing the risk of distant metastasis. There are currently some selected skull base diseases for which induction/neoadjuvant treatment has demonstrated to be a valuable first-choice treatment option. In particular, in sinonasal undifferentiated carcinomas, the entity responding to induction chemotherapy has demonstrated to be an important predictor of the success of subsequent locoregional treatment (surgery vs. chemoradiation) [19]. Moreover, the use of neoadjuvant chemotherapy has been proposed for orbit preservation attempts in advanced sinonasal cancers that have a critical relationship with the orbit when committed to surgical management [20,21].

In addition to malignant tumors requiring highly demanding multidisciplinary approaches, benign skull base conditions also pose a challenge. Osseous diseases, such as fibrous dysplasia [22] and osteomas [23], may significantly disrupt skull base anatomy and cause compression symptoms in the surrounding critical structures, including the orbit, cranial nerves, and vessels. These diseases’ surgical treatment can be extremely challenging, requiring endoscopic or combined endoscopic–external approaches and may be delayed until the disease becomes symptomatic. However, when symptoms are present, patients should be treated promptly to avoid functional sequelae. Other histologically benign conditions, such as meningiomas [24] involving the skull base, may present with severe symptoms and can progress so as to cause severe morbidity due to cranial nerve dysfunction. On the other hand, surgery itself, whether transnasal endoscopic, transcranial, or transtemporal, may result in potential morbidity; thus, in the case of benign conditions, the potential risks related to disease progression and those related to treatment should be accurately balanced. As a result, the main challenges concerning skull base surgery for benign conditions reside in minimizing invasiveness, reducing the risk of complications, and identifying the most appropriate timing for intervention.

Lastly, inflammatory diseases of the skull base deserve special attention. Osteomyelitis represents a rare but severe condition that is potentially associated with life-threatening complications [25]. The gateway for infection is often related to direct communication with the sinonasal district or the mastoid–middle ear complex but may also derive from the evolution of a necrotizing external otitis [26]. The management of osteomyelitis requires the long-term, intravenous administration of antibiotics, usually prescribed in collaboration with an infectious disease specialist, along with surgical debridement [25]. Similar considerations also apply to skull base osteoradionecrosis following radiation therapy in the same region [27]. In this case, in addition to surgical debridement and antibiotic coverage, hyperbaric oxygen therapy and vascular carrier flaps may be beneficial [28].

This Special Issue of the *Journal of Clinical Medicine* entitled “State of the Art-Treatment of skull base diseases” aims to compose a comprehensive picture of the state of the art regarding the multidisciplinary management of skull base diseases in all its complexity, while simultaneously outlining the emerging trends and future development perspectives in this intriguing field of research.

## Figures and Tables

**Figure 1:**
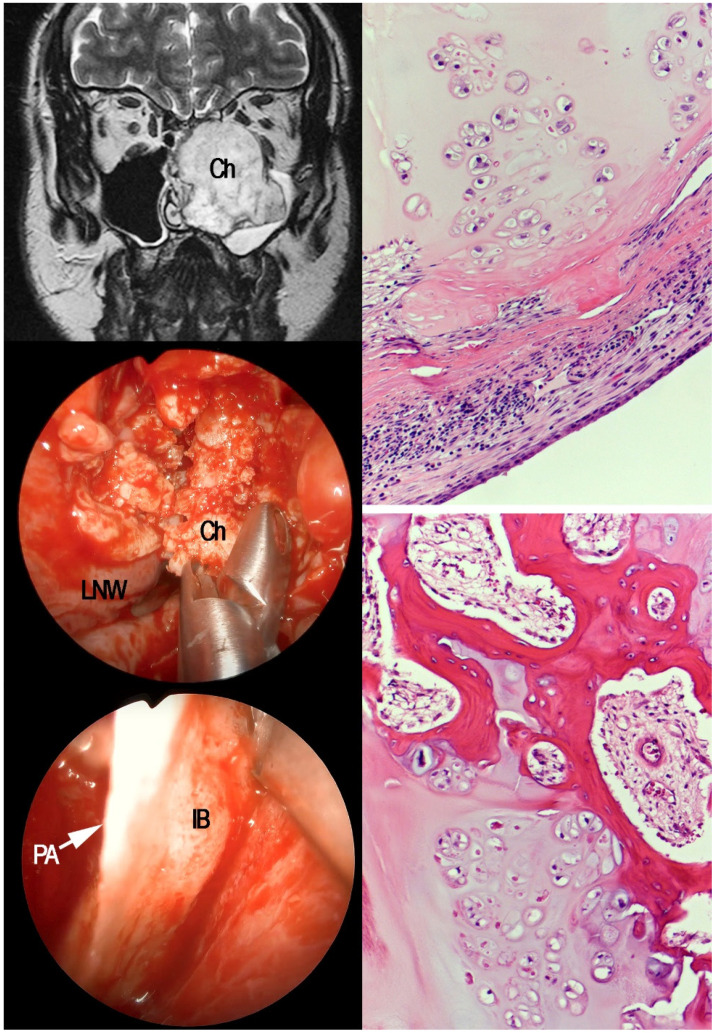
Radiological, endoscopic, and pathologic appearance of an endoscopically resected, well-to-moderately differentiated, sinonasal chondrosarcoma (Ch). The upper pathologic image shows the submucosal growth pattern of chondrosarcoma; the lower pathologic image shows the permeative intra-osseous growth pattern of the lesion. IB, grossly infiltrated bone; LNW, lateral nasal wall (right side); PA, piriform aperture.

**Figure 2:**
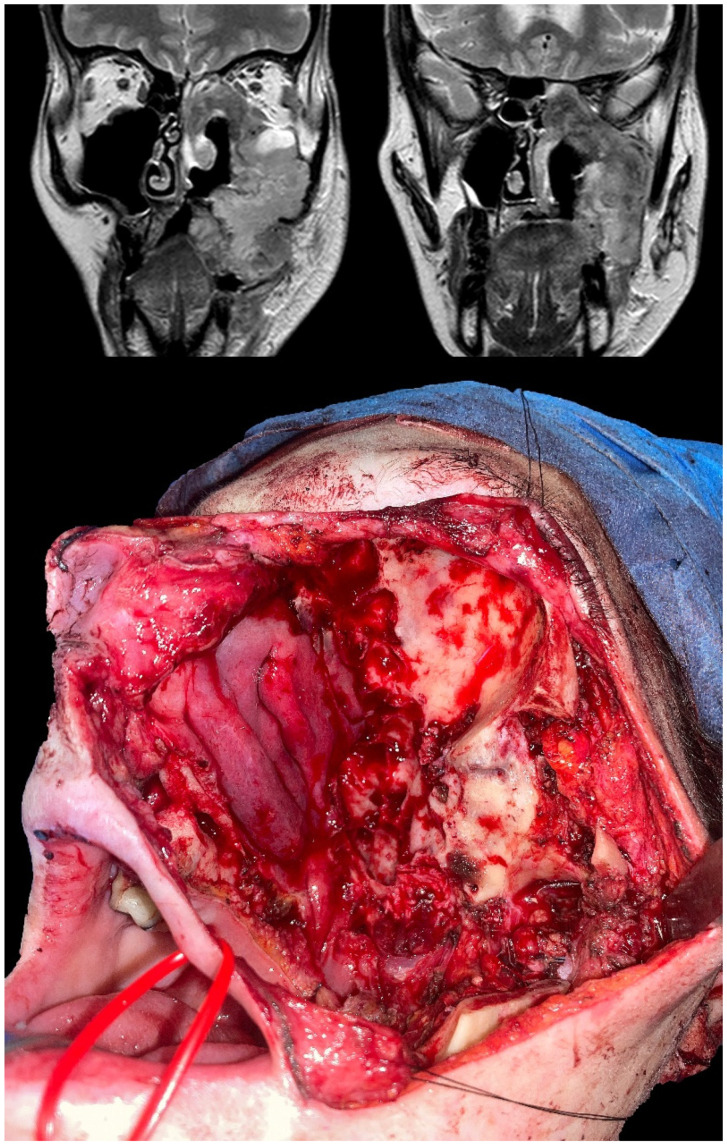
Radiological appearance of a maxillary, poorly differentiated leiomyosarcoma and intraoperative picture taken after ablation. The anterior and middle skull base’s outer surfaces were completely exposed and partially removed as part of the resection.

**Figure 3:**
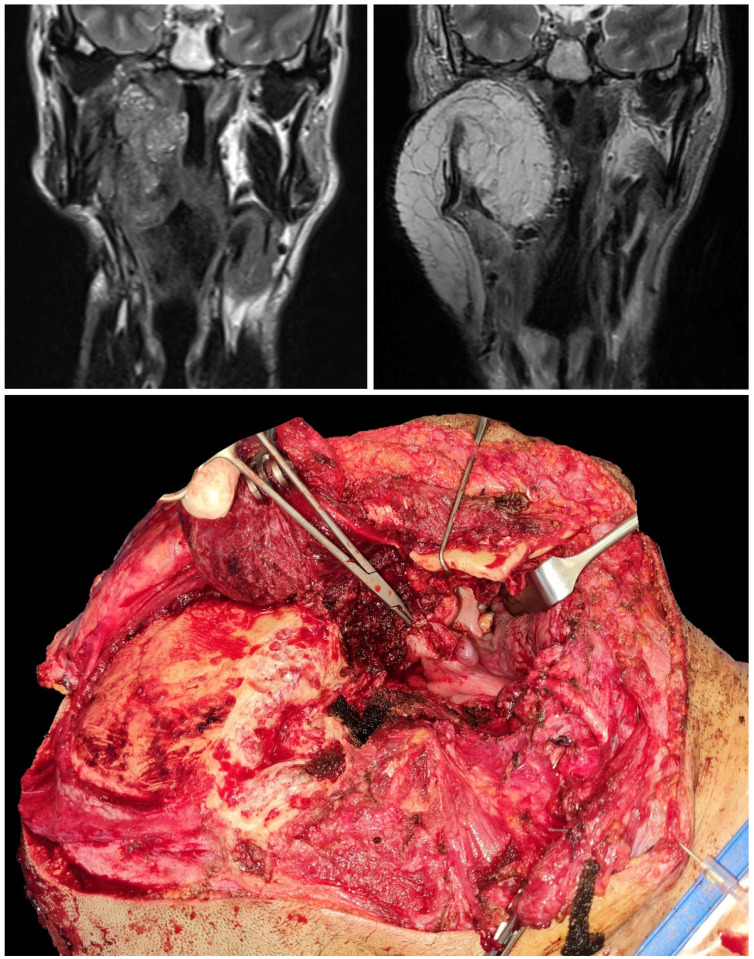
Pre-operative (upper left image) and post-operative (upper right image) magnetic resonance imaging of a patient affected by relapsing NUT carcinoma of the right parotid. Intraoperative (lower image) appearance of the surgical field after the ablation phase, which included a temporo-parotid resection [16] extended to the parapharyngeal space, masticator space, lateral nasopharyngeal and oropharyngeal walls, and middle cranial base.

**Figure 4:**
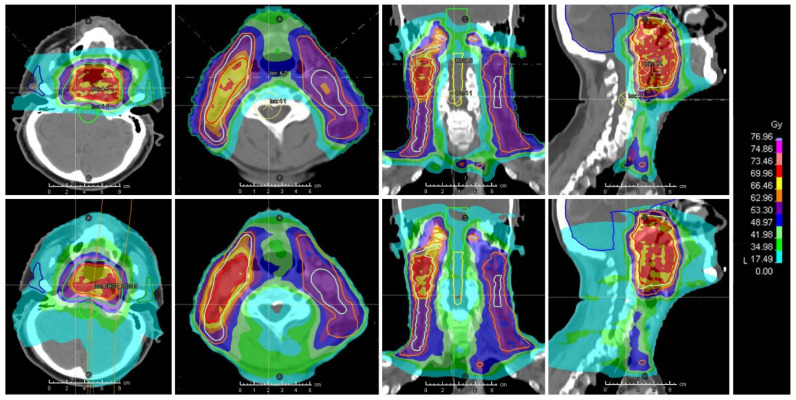
Comparison of dose distribution between a proton beam-based (upper row) and photon-based (lower row) intensity-modulated radiotherapy plan in a patient affected by nasopharyngeal carcinoma. Courtesy of Dr. Ester Orlandi, Dr. Alessandro Vai (National Center for Oncological Hadrontherapy, Pavia, Italy), and Dr. Nicola A Iacovelli (National Cancer Institute, Milan, Italy).

**Figure 5:**
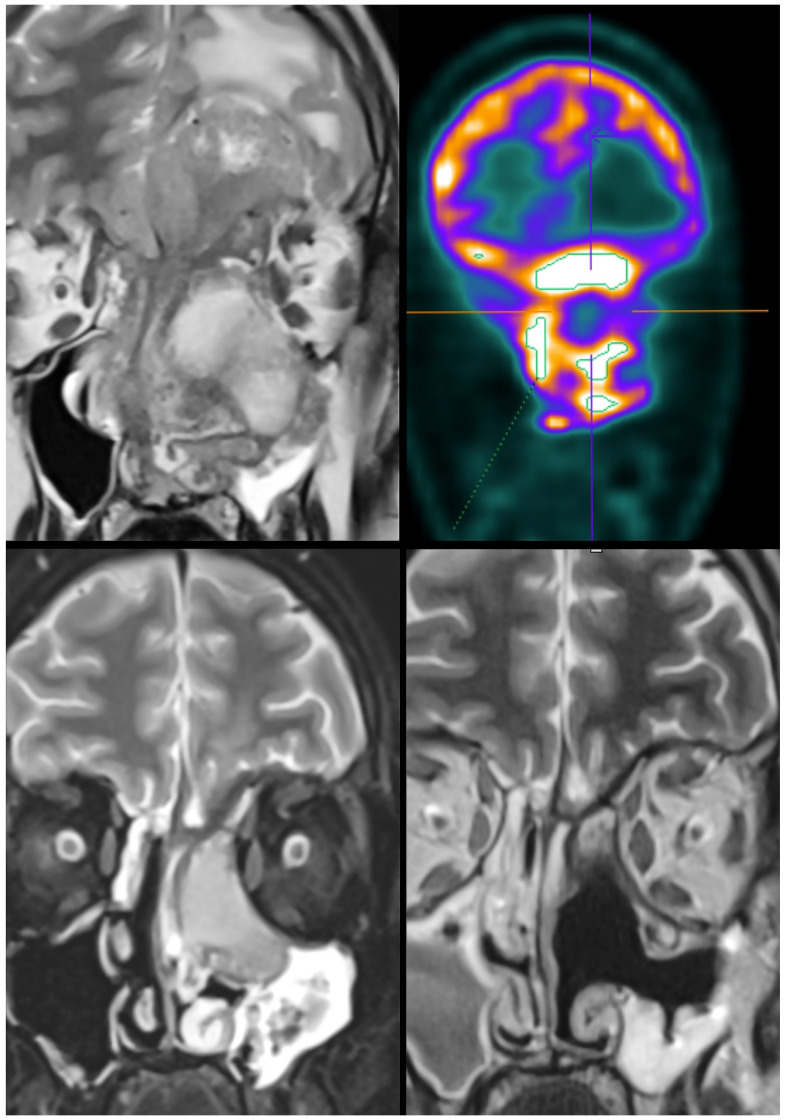
Non-surgical treatment of a sinonasal, undifferentiated carcinoma. The upper images show magnetic resonance and positron emission tomography with ^18^F-fluorodeoxyglucose of a patient affected by a large sinonasal, undifferentiated carcinoma invading the orbit and intracranial space and causing brain edema. The lower left image shows partial response of the tumor after two cycles of neoadjuvant chemotherapy with the TPF (taxane, cisplatin, and fluorouracil) regimen. The lower right image shows complete response after definitive chemoradiation with proton beam therapy and weekly administration of cisplatin.

## Data Availability

No new data were created or analyzed in this study. Data sharing is not applicable to this article.
